# Quantitative evaluation method of stroke association based on multidimensional gait parameters by using machine learning

**DOI:** 10.3389/fninf.2025.1544372

**Published:** 2025-02-12

**Authors:** Cheng Wang, Zhou Long, Xiang-Dong Wang, You-Qi Kong, Li-Chun Zhou, Wei-Hua Jia, Pei Li, Jing Wang, Xiao-Juan Wang, Tian Tian

**Affiliations:** ^1^Jinan Zhougke Ubiquitous-Intelligent Institute of Computing Technology, Jinan, China; ^2^Shandong Academy of Intelligent Computing Technology, Shandong Institutes of Industrial Technology (SDIIT), Jinan, China; ^3^Ningbo Institute of Information Technology Application CAS, Ningbo, China; ^4^Bejing Key Laboratory of Mobile Computing and Pervasive Device, Beijing, China; ^5^Institute of Computing Technology (ICT) Chinese Academy of Sciences (CAS), Beijing, China; ^6^Department of Neurology, Beijing Chao-Yang Hospital, Capital Medical University, Beijing, China; ^7^Department of Neurology, Beijing Shijingshan Hospital, Shijingshan Teaching Hospital of Capital Medical University, Beijing, China; ^8^General Practice Department, Beijing Chao-Yang Hospital, Capital Medical University, Beijing, China

**Keywords:** quantitative evaluation, gait parameters, stroke, NIHSS, machine learning

## Abstract

**Objective:**

NIHSS for stroke is widely used in clinical, but it is complex and subjective. The purpose of the study is to present a quantitative evaluation method of stroke association based on multi-dimensional gait parameters by using machine learning.

**Methods:**

39 ischemic stroke patients with hemiplegia were selected as the stroke group and 187 healthy adults from the community as the control group. Gaitboter system was used for gait analysis. Through the labeling of stroke patients by clinicians with NIHSS score, all gait parameters obtained were used to select appropriate gait parameters. By using machine learning algorithm, a discriminant model and a hierarchical model were trained.

**Results:**

The discriminant model was used to distinguish between healthy people and stroke patients. The overall detection accuracy of the model based on KNN, SVM and Randomforest algorithms is 92.86, 92.86 and 90.00%, respectively. The hierarchical model was used to judge the severity of stroke in stroke patients. The model based on Randomforest, SVM and AdaBoost algorithm had an overall detection accuracy of 71.43, 85.71 and 85.71%, respectively.

**Conclusion:**

The proposed stroke association quantitative evaluation method based on multi-dimensional gait parameters has the characteristics of high accuracy, objectivity, and quantification.

## Graphical abstract

Diagram of quantitative evaluation method of stroke association based on gait parameters.
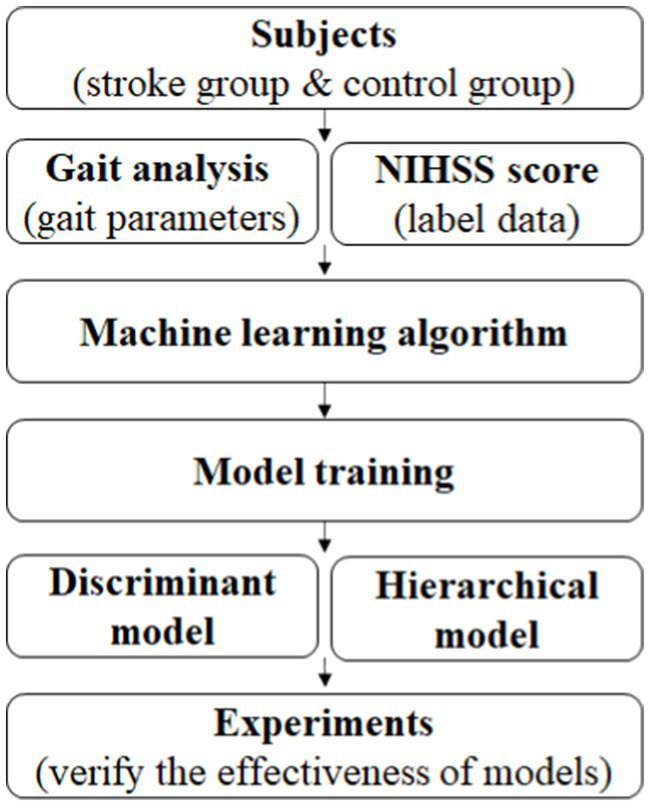

## 1 Introduction

Stroke has a high incidence rate, mortality, disability, and recurrence rate, ranking among the leading causes of death among residents for many years (Wan and Dong, 2017; Wang W. et al., 2017). More than 60% of stroke patients still have different degrees of neurological dysfunction after treatment (Wang L. D. et al., 2018), which seriously affects the quality of life of patients. The main reasons for the delay before hospitalization of stroke patients were that they could not quickly identify the signs of stroke or did not give first aid at the first time. Early warning of the occurrence or recurrence of stroke and active emergency measures are of great significance for reducing the disability rate and mortality rate of stroke and improving the prognosis of patients (Nagao et al., 2020) Patients with hemiplegia need to assess the motor function of lower limbs, which is conducive to the formulation of rehabilitation plans, and the monitoring of limb function during the rehabilitation process, to adjust the treatment plan in time. NIHSS is the most widely used clinical and experimental stroke functional examination scale, which is recognized as reliable, effective, and sensitive (Nagao et al., 2020; Khalil and Lahoud, 2020). It can help clinicians accurately assess and communicate with each other about the neurological deficit of stroke patients and guide patients to make long-term rehabilitation and nursing plans (Wang Y. X. et al., 2016; Wu et al., 2019; Jauch et al., 2013). However, NIHSS contains many contents, which is complex and professional and cannot be mastered by ordinary patients outside the hospital. Therefore, for stroke evaluation, a simple, quantitative, objective, and rapid method or system is needed.

## 2 Related work

With the development of science and technology, gait analysis technology has been gradually applied to the gait research of stroke (Wan et al., 2014; Aoki et al., 2013; Estrada-Barranco et al., 2022; Mizuta et al., 2022; Chow and Stokic, 2021). At present, gait analysis in clinic is mostly used to reveal that there are obvious differences in gait of stroke patients in many aspects. By determining gait characteristics (gait parameters) of stroke patients, it can effectively evaluate the effect of rehabilitation quality of stroke patients (Seok et al., 2021; Hussain and Park, 2021; Titus et al., 2018; Hu et al., 2009). In addition, there are a small number of artificial intelligence studies on gait analysis of stroke patients, such as distinguishing whether it is stroke according to the symmetry, regularity, and stability of gait (Kim et al., 2022; Krawczyk et al., 2012), and there are also studies on classification and evaluation of gait of stroke patients (Li et al., 2019; Andrea et al., 2016; Kaczmarczyk et al., 2009; Cui et al., 2018). Almost no artificial intelligent studies of stroke are based on more comprehensive spatial–temporal characteristics (parameters) of gait.

In this paper, combined with the NIHSS score for stroke in clinic and gait analysis, the study is on quantitative evaluation and analysis of stroke association using machine learning method. Since the gait analysis system is wearable and easy to operate simply, the objective of the study is to warn the occurrence or recurrence of stroke outside the hospital, and to assist doctors in quantitative, objective and simple identification and grading of stroke degree in the hospital. Specifically, as it is shown in graphical abstract, through the labeling of stroke patients by clinicians with NIHSS score, all gait parameters obtained from gait analysis system were used to select appropriate gait parameters. By using machine learning algorithm, a discriminant model for distinguishing between normal healthy people and stroke patients, and a hierarchical model for judging the severity of stroke in stroke patients were trained. Finally, experiments were conducted to evaluate the effectiveness of the two trained models.

## 3 Materials and methods

### 3.1 Participants of study

39 ischemic stroke hemiplegic patients admitted to Beijing Chao-Yang Hospital, Capital Medical University from June 2018 to October 2018 were selected as the stroke group, including 24 males and 15 females, aged (63.4 ± 14.5) years, with a height of (169.3 ± 7.1) cm and a weight of (77.5 ± 7.5) kg; 187 healthy adults from the community served as the control group, including 68 males and 119 females, aged (47.4 ± 12.4) years, with a height of (165.2 ± 7.4) cm and a weight of (69.7 ± 17.6) kg. Inclusion criteria: ① patients with gait abnormality caused by acute stroke; ② the muscle strength of the affected limb is grade IV or above, and can walk independently; ③ the cognitive function is normal and can cooperate with the gait parameter acquisition process. Exclusion criteria: ① vestibular / cerebellar dysfunction and other lower limb muscle, bone and nervous system diseases that can cause walking disorders; ② Serious systemic diseases such as heart, lung, liver, and kidney dysfunction. Inclusion criteria of the control group: no nervous system, lower limb muscle, bone and other diseases, no serious cardiopulmonary diseases. The study was approved by the institutional review board of Beijing Chao-yang Hospital, Beijing China, and was conducted in accordance with the Declaration of Helsinki. All participants have signed informed consent forms. The study protocol was submitted and approved by the Research Ethics Committee of the Beijing Chao-Yang Hospital (the number is 2018-sci-153). Institutional Review Board reference date is 2018-6-11.

### 3.2 Test tools of gait analysis

The gait analysis equipment used in this study was the Gaitboter gait analysis system developed by the Institute of computing technology, Chinese Academy of Sciences. The system includes a wearable gait acquisition device and corresponding gait analysis software that integrates motion sensors, plantar membrane pressure and sound. Gaitboter is a sports shoe with a multi-sensor fusion gait data acquisition circuit built in. The sensors include accelerometers, gyroscopes, and plantar membrane pressure sensors (sampling rate is 80 Hz) and micro microphones (sampling rate is 4,000 Hz). The mobile phone or tablet with gait analysis software can collect and analyze the gait data obtained by the acquisition device through Bluetooth. Previous experiments show that due to the fusion of multiple sensor information, the accuracy of gait space–time parameters measured by the system used in this paper is higher than that measured by the system using only inertial sensors such as acceleration and gyroscope (Wang C. et al., 2016; Wang C. et al., 2017) at the same time, it has good sensitivity and reliability (Kong et al., 2018).

### 3.3 Test methods of gait analysis

① The experimental operator was an experienced doctor who explained the experimental process and precautions to the subjects before the test; ② The subjects wore comfortable and light clothes and chose wearable gait analysis equipment of appropriate size; ③ Choose a 10 m long and 3 m wide walkway with sufficient light, flat ground and no obstacles as the gait analysis walkway; ④ During the test, the subjects were required to walk in a straight line at a uniform speed with their own gait without interference. According to the actual situation of the subjects, tried to let the subjects walk more times, that is, different subjects walked different times, and each walking process covered about 2–50 steps. Gait information collected by wearable devices was transmitted to mobile phones or tablets through Bluetooth. The gait parameters of subjects were analyzed automatically. Before the formal test, the subjects needed to walk on the walkway twice to adapt to the environment.

## 4 Quantitative evaluation method of stroke association based on multidimensional gait parameters

**Figure 1:**
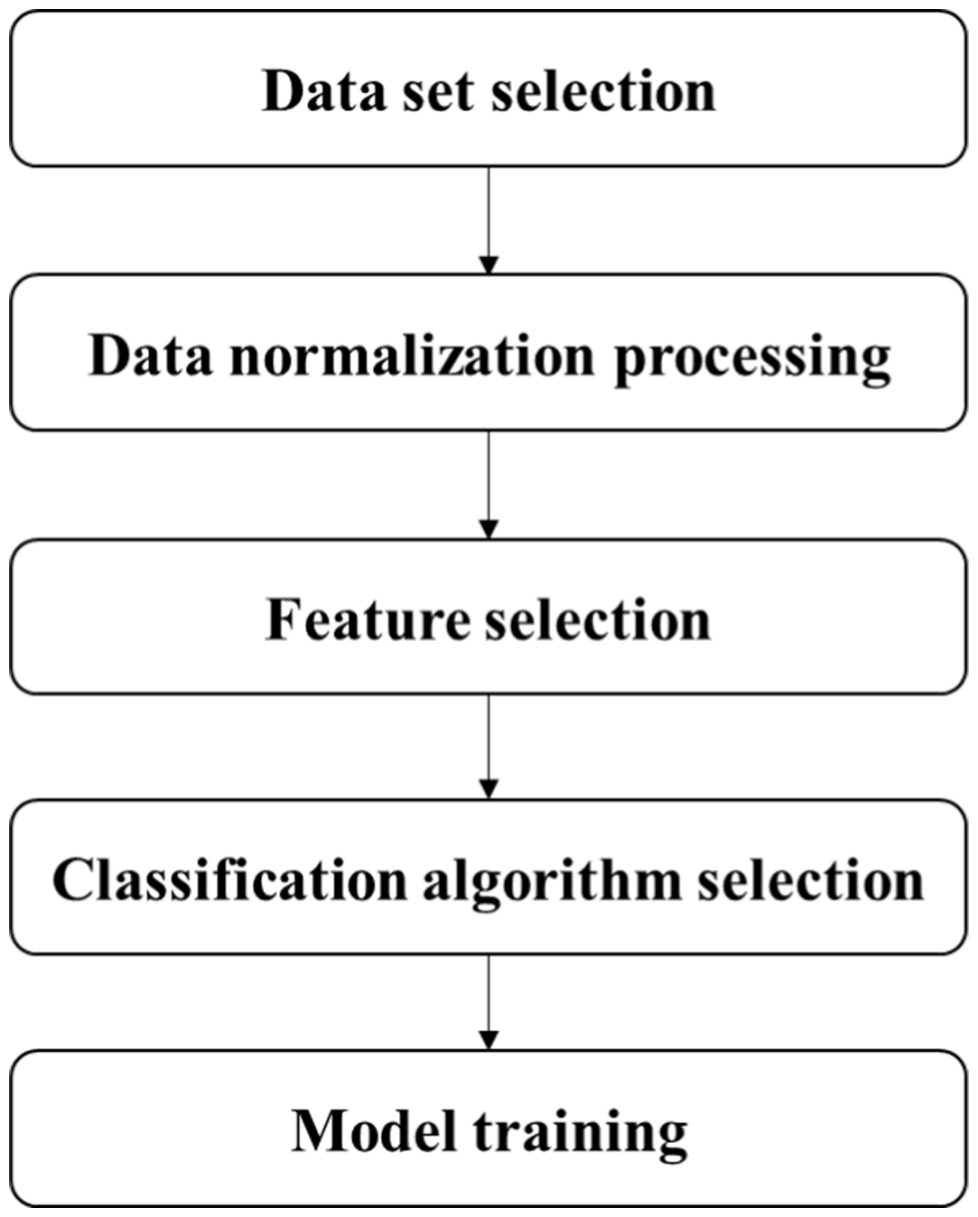
Diagram of stroke gait analysis model establishment process.

The subjects in the stroke group were scored by experienced doctors according to the NIHSS to assess the severity of stroke. NIHSS consists of 11 evaluation indexes, including consciousness level, gaze, visual field, facial paralysis, upper limb movement, lower limb movement, etc. The higher the score, the worse the function. The minimum score is 0 and the maximum score is 42. In previous studies, Kongyouqi et al. (Kong et al., 2018), Gaitboter gait analysis system was used to analyze and detect the gait of stroke patients and healthy control group. It was found that compared with the control group, the stride of the stroke group was shorter, the gait frequency and speed were decreased, and the swing phase time, support phase and bipedal support time were prolonged (*p* < 0.01). Based on this conclusion, as it is shown in graphical abstract, this paper presents an intelligent gait analysis method for stroke: firstly, appropriate gait features were selected from all the existing gait features (gait parameters); secondly, stroke patients were labeled with NIHSS score by clinicians; finally a classification model for stroke patients (hereinafter referred to as the discrimination model) and a classification model for stroke severity (hereinafter referred to as the hierarchical model) through machine learning algorithm were trained. The establishment steps of the two models were basically divided into data set selection, data normalization processing, feature selection, classification algorithm selection and model training, which are described in detail below. Figure 1 shows the flow diagram of model construction.

### 4.1 Data set selection

**Gait characteristics**: A high-precision gait detection and parameter calculation method were adopted (Wang C. et al., 2016; Wang C. et al., 2017; Wang C. et al., 2018). A total of 32 gait characteristic parameters were calculated. According to the parameter categories, they were classified as follows (Table 1):

**Table 1:** Gait characteristic parameters (Tian et al., 2021).

Type	Gait parameters
Temporal parameter characteristics (8)	left swing phase\right swing phase left stance phase\right stance phase left time\right time left double time\right double time
Spatial parameter characteristics (24)	left stride\right stride left step frequency\right step frequency left step speed\right step speed left foot rotation\right foot rotation left off ground angle\right off ground angle left ground angle\right ground angle left sagittal min angle\right sagittal min angle left sagittal max angle\right sagittal max angle left coronal min angle\right coronal min angle left coronal max angle\right coronal max angle left cross min angle\right cross min angle left cross max angle\right cross max angle

**Figure 2:**
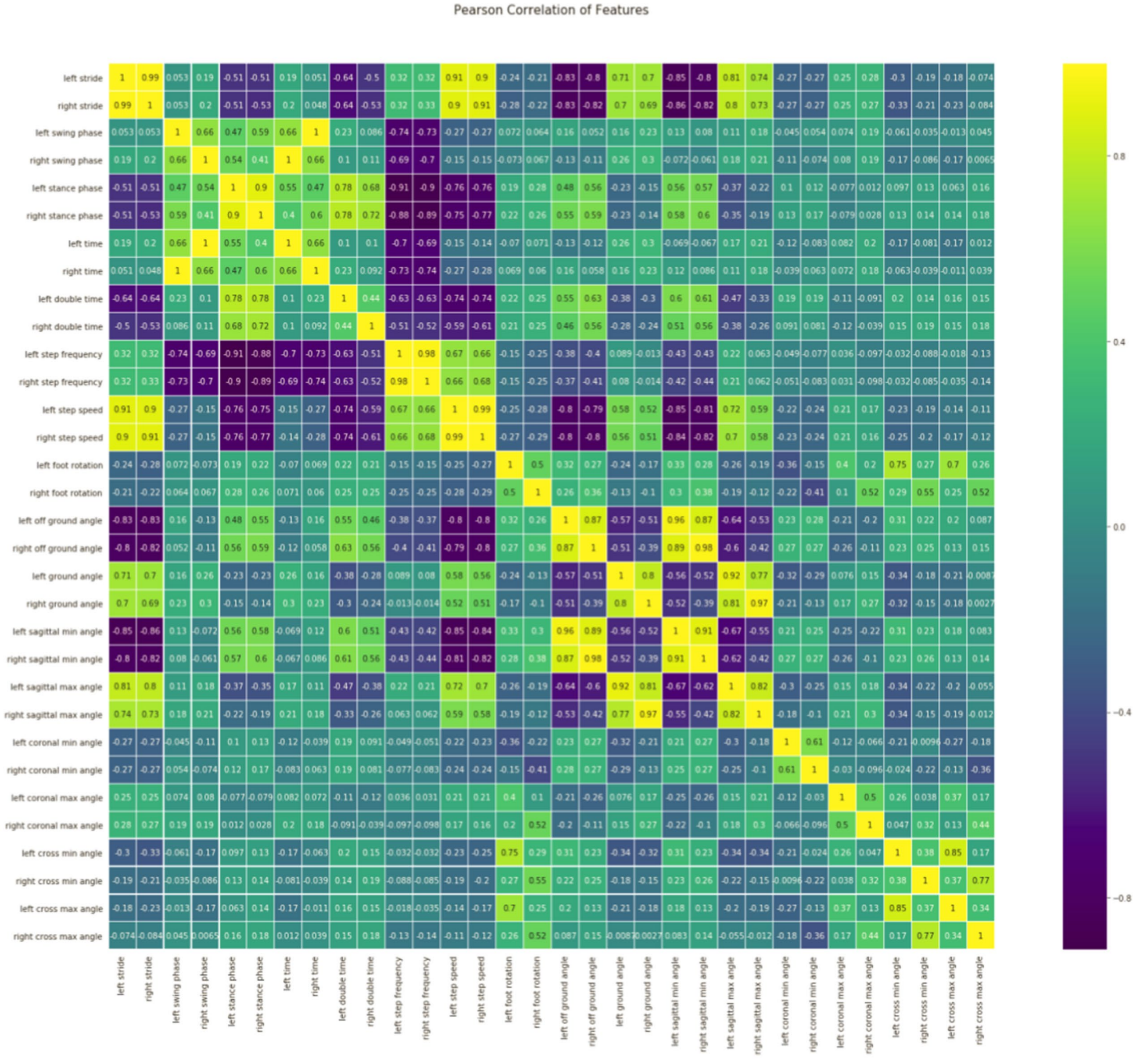
Relevance analysis of features.

**Feature correlation analysis**: As it is shown in Figure 2, stride is positively correlated with step speed, and stride is also positively correlated with ground angle and the sagittal max angle; Step speed is positively correlated with the sagittal max angle; Left time is positively correlated with right swing phase; The cross min (max) angle is positively correlated with foot rotation; Ground angle is also positively correlated with step speed; The left (right) of cross min (max) angle is positively correlated with the left (right) of cross max (min) angle, which is consistent with the situation of normal gait walking. These gait feature parameters will be used as the features of our next model training data set, which is divided into training set and test set.

As we know, by using all 32 feature parameters including the highly correlated ones, the machine learning models can be biased, therefore, reducing the features redundancy before using all 32 feature parameters in machine learning models is important. In addition, too many features may also lead to over-fitting and more calculation, and too few features may lead to under-fitting. In this paper, cross-validation was used to select the optimal feature combination and eliminate redundant features and aimed at evaluating the impact of different feature subsets on model performance (In the section Feature Selection, there is a detailed introduction).

**Data label**: For the label of the data in the data set (including training set and test set), we adopted the NIHSS score which is used to assess the severity of stroke patients. The NIHSS consists of 11 evaluation indexes, each of which has a score range of 0–4 points. A score of 0 indicates that the evaluation index has normal function, and the higher the score, the worse the function. After all the 11 evaluation indicators are evaluated, the total score of NIHSS will be generated. The minimum score is 0 and the maximum score is 42 (Hage, 2011), as it is shown in Table 2. Experienced clinicians score stroke patients in the data set through the NIHSS mentioned above, and it is data label.

**Table 2:** Table of comparing between NIHSS scores and stroke severity.

Score	Stroke severity
0	None
1–4	Slight
5–15	Moderate
16–20	Moderate to severe
21–42	Serious

### 4.2 Data normalization processing

The gait characteristic parameters described above represent different physical meanings, and the numerical magnitude is also very different. Therefore, before model training, we need to normalize all the data, otherwise, especially the features with large data values will have a great impact on model training (Duda, 2001). The normalization method is to quantitatively convert the values, that is, the values corresponding to each parameter are mapped to a small range. Here we take (−1, 1), and the normalization calculation formula is as follows in Equation 1:


(1) $$x^{\prime }=-1+\frac{2x}{x_{\text{max}}-x_{\text{min}}}$$

x is the value of the original data, $x^{\prime }$ is the scaled value, $x_{\text{max}}$ is the maximum value and $x_{\text{min}}$ is the minimum value in the data.

### 4.3 Feature selection

**Figure 3:**
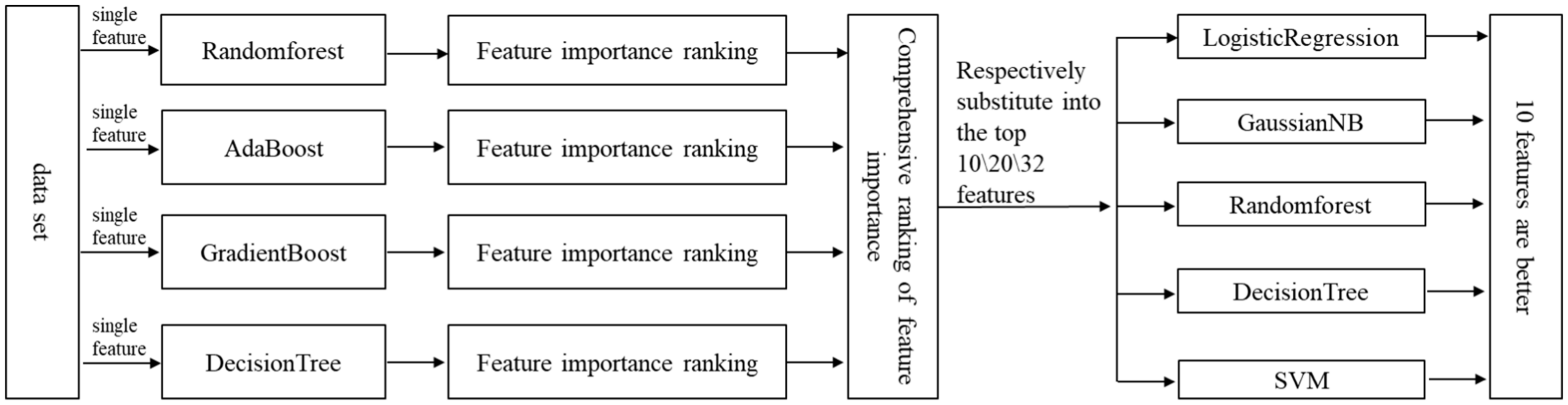
Process of feature selection based on ensemble-learning-based methods.

Feature selection is very important for the final training model. Improper feature selection leads to poor classification performance of the trained model. Even if the feature selection is appropriate, the number (dimension) of features will also have a great impact on the classification performance of the final training model. Too many features may lead to over-fitting and more calculation, and too few features may lead to under-fitting. The feature selection methods used in this study is a kind of ensemble-learning-based methods that aim to construct a group of feature subsets from several different algorithms, and then produce an aggregated result out of the group. In this way, the instability and perturbation issues of single algorithm can be alleviated, and also, the subsequent learning (model training) tasks can be enhanced (Li et al., 2017). In this paper, we use Randomforest (Ho, 1995; Ho, 1998; Hastie et al., 2001; Mariana and Lucian, 2016; Biau and Scornet, 2015), AdaBoost (Joglekar, 2016; Kégl, 2013; Collins et al., 2002), GradientBoosting (Friedman, 2001; Mason et al., 1999; Zhu, 2016; Ayyadevara, 2018), DecisionTree (Rokach and Maimon, 2008; Freund and Mason, 2002; Fürnkranz, 2017). Although a comparison of Randomforest-based feature selection methods (Speiser et al., 2019) presented that the method VSURF (Genuer et al., 2015) & Boruta (Kursa and Rudnicki, 2010) implemented in the R package are preferable, combined to the characteristics of this study, the reason for not choosing this method is: (a) it is a feature selection method obtained by a single method (not ensemble-learning method), without considering the instability and perturbation issues of a single algorithm; (b) it also does not consider the coherence of subsequent learning (model training) tasks, such as the inconvenience of integration caused by the different environments of R language and the overall system implementation (Python); (c) our paper adopts an ensemble-learning-based method, which can improve the robustness of feature selection by integrating multiple model algorithms; (d) the method used in our paper integrates the feature importance of multiple model algorithms to fully control the process of feature selection, and it has good flexibility, controllability and interpretability. Specifically, the algorithm in our study filters the features and selects the most important features. The idea of this method is to directly use the machine learning model to select features, establish prediction models for each individual feature and response variable, and use cross-validation to select models. The specific steps and processes are shown in Figure 3.

According to Figure 3, the typical process of feature selection is to first determine the selection criteria and then determine which features to select. Here, the selection criteria is the feature numbers/dimensions, and the determination of which features should be selected is the highest ranking of feature importance according to the numbers/dimensions. Feature numbers (dimensions) are gotten as follows: Considering that there are 32 feature parameters in the data set, in order to avoid the problems of over-fitting and under-fitting caused by too much or too little feature selection, we selected the top 10 features, 20 features and 32 features (all features) from the ranking(feature sets were manually divided into small, medium, and large three different levels subsets: 10/20/32) according to their importance, and used LogisticRegression (Cox, 1958; Liu et al., 2009; Lin et al., 2007), GaussianNB (Long et al., 2012; James and Vimina, 2022), DecisionTree, Randomforest, SVM (Vapnik, 1998; Zheng et al., 2008; Tong and Chang, 2001) algorithm for cross-validation to obtain their accuracy. Then we compared the accuracy of 10 features, 20 features and 32 features in the case of each algorithm, and further comprehensively judged the number of features selected by the final classifier.

In this case, final features selection are as follows: The number of features has been determined above, so we need to determine the specific features. The ranking of feature importance was selected by the ensemble of four different models/algorithms: Randomforest, AdaBoost, Gradientboosting and Decisiontree. According to the number of previously determined features, we selected the corresponding number of features with the highest ranking according to their importance, and finally formed a feature vector.

**Figure 4:**
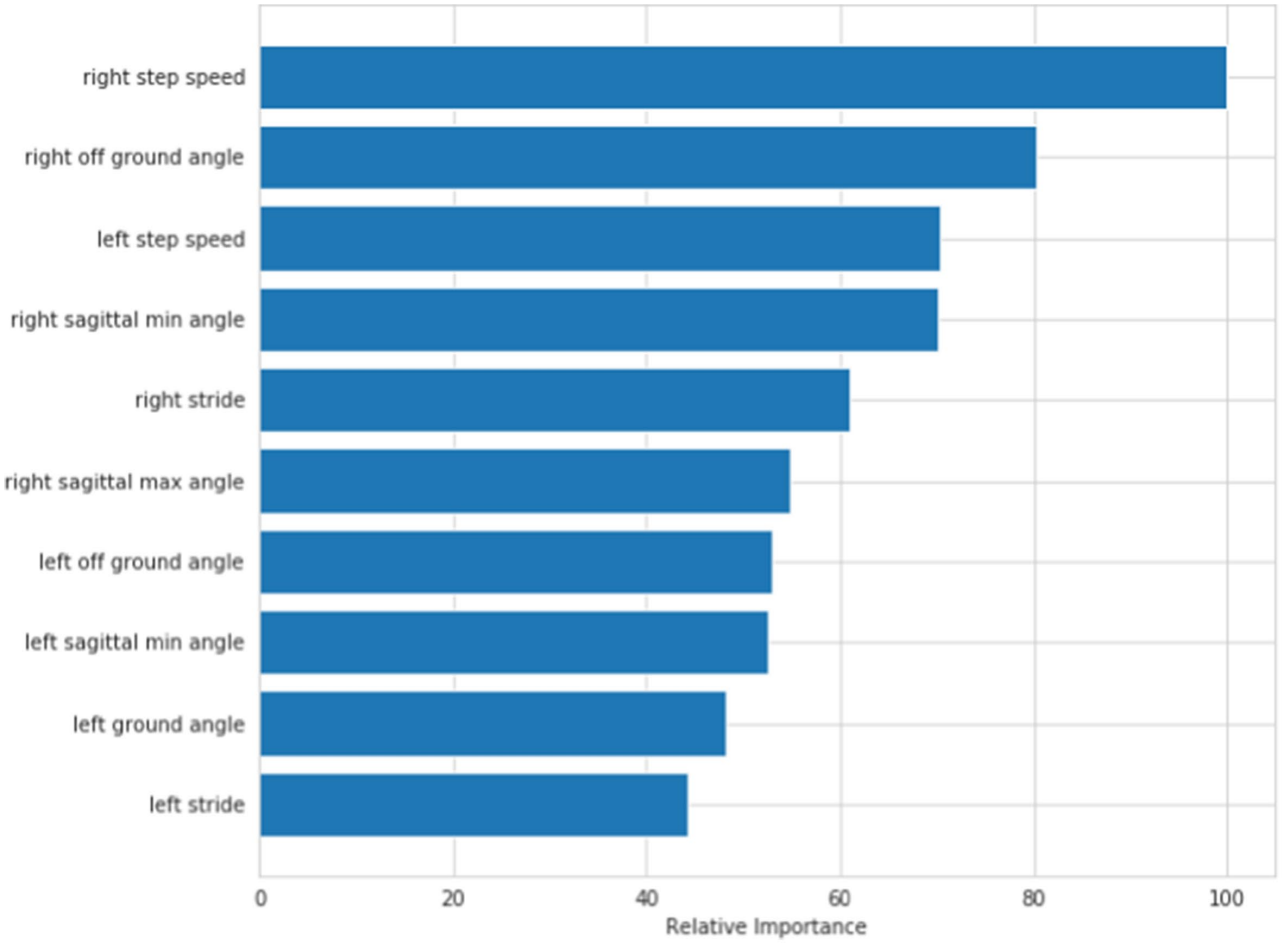
Discriminant model: order of feature’s importance.

**Discriminant model**: According to the above feature selection algorithm, 10 features, 20 features and 32 features were selected for cross-validation with 5 common algorithms, and their corresponding accuracy is shown in Table 3. It can be seen from the table that when 10 features are selected, the classifier has better classification effect and less calculation. Figure 4 shows the visual sorting of feature importance selected by the ensemble of four different models/algorithms: Randomforest, AdaBoost, Gradientboosting and Decisiontree algorithms (the abscissa is the relative importance of features). In this way, the 10 features we finally selected form a feature vector: right step speed, left step speed, right off ground angle, left off ground angle, right sagittal min angle, left sagittal min angle, right stride, left stride, right sagittal max angle, left sagittal max angle.

**Table 3:** Discriminant model: table of comparing for features selection.

Algorithm	10 features	20 features	32 features
LogisticRegression	90.42%	87.19%	82.09%
GaussionNB	88.46%	86.49%	87.19%
DecisionTree	84.09%	82.67%	78.76%
Randomforest	89.75%	88.46%	89.15%
SVM	89.77%	89.77%	88.55%

**Hierarchical model**: According to the feature selection algorithm, 10 features, 20 features and 32 features are selected for cross-validation with five common algorithms, and their corresponding accuracy is shown in Table 4. It can be seen from the table that when 10 features are selected, the classifier has better classification effect and less calculation.

**Table 4:** Hierarchical model: table of comparing for features selection.

Algorithm	10 features	20 features	32 features
LogisticRegression	44.30%	43.50%	43.59%
GaussionNB	47.98%	47.51%	46.59%
DecisionTree	55.75%	54.61%	52.97%
Randomforest	67.10%	66.19%	62.03%
SVM	64.25%	63.68%	64.11%

**Figure 5:**
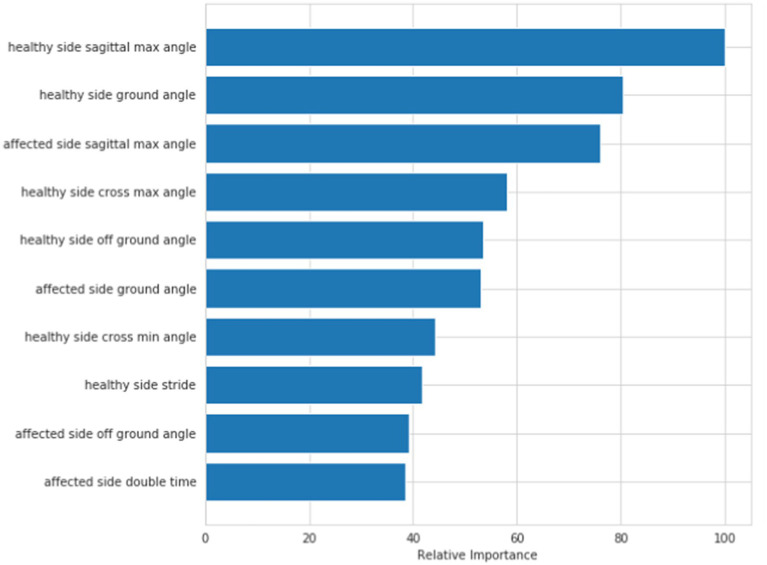
Hierarchical model: order of feature’s importance.

Figure 5 shows the visual sorting of feature importance selected by the ensemble of four different models/algorithms: Randomforest, AdaBoost, Gradientboosting and Decisiontree algorithms (the abscissa is the relative importance of features). In this way, the 10 features finally selected form a feature vector: healthy side ground angle, affected side ground angle, healthy side off ground angle, affected side off ground angle, healthy side cross max angle, affected side cross max angle, healthy side stride, affected double time, healthy side sagittal max angle, affected side sagittal max angle.

### 4.4 Classification algorithm selection

After determining the features of model training, it is necessary to select a classification algorithm. We selected the classification algorithm of the model through cross-validation. Specifically, we selected nine common classification algorithms and calculated the performance indicators of each classification algorithm. Each algorithm performed 5-fold cross-validation calculations, calculated the prediction accuracy for five times, and obtained the prediction accuracy of the algorithm (the average value of 5 times, and the average accuracy is one of the commonly used evaluation indicators). We compared the corresponding accuracy values obtained in the case of each algorithm, and finally selected the algorithm with higher accuracy value as our model training algorithm.

**Discriminant model**: According to the determination method mentioned above, each algorithm’s accuracy value, standard deviation and confidence interval are shown in Table 5. Through comparison, the following three algorithms are selected for classifier modeling: KNN, SVM and Randomforest.

**Table 5:** Discriminant model: comparing between different algorithms for algorithm’s selection.

Algorithm	Accuracy±standard deviation	95% confidence interval
KNN (Altman, 1992; Wong et al., 2009; Soucy and Mineau, 2001)	0.8978 ± 0.0711	(0.8096, 0.9860)
Perceptron (Freund and Schapire, 1999; Gallant, 1990; Ruck, 1990)	0.8530 ± 0.0504	(0.7905, 0.9156)
AdaBoost	0.8589 ± 0.0333	(0.8176, 0.9002)
Stochastic Gradient Descent (Robbins and Monro, 1985)	0.8405 ± 0.0674	(0.7569, 0.9242)
LogisticRegression	0.8595 ± 0.0647	(0.7792, 0.9398)
SVM	0.9105 ± 0.0510	(0.8471, 0.9738)
DecisionTree	0.8204 ± 0.0636	(0.7415, 0.8993)
Randomforest	0.9105 ± 0.0229	(0.8821, 0.9389)
GradientBoosting	0.8268 ± 0.0601	(0.7522, 0.9014)

**Hierarchical model**: According to the determination method mentioned above, each algorithm’s accuracy value, standard deviation and confidence interval are shown in Table 6. Through comparison, the following three algorithms are selected for classifier modeling: SVM, Randomforest and AdaBoost.

**Table 6:** Hierarchical model: comparing between different algorithms for algorithm’s selection.

Algorithm	Accuracy ± standard deviation	95% confidence interval
KNN (Altman, 1992; Wong et al., 2009; Soucy and Mineau, 2001)	0.6821 ± 0.2295	(0.3972, 0.9670)
Perceptron (Freund and Schapire, 1999; Gallant, 1990; Ruck, 1990)	0.6040 ± 0.1949	(0.3620, 0.8459)
AdaBoost	0.6981 ± 0.1026	(0.5706, 0.8255)
Stochastic Gradient Descent (Robbins and Monro, 1985)	0.6101 ± 0.1212	(0.4596, 0.7606)
LogisticRegression	0.4488 ± 0.1028	(0.3212, 0.5765)
SVM	0.6869 ± 0.1216	(0.5359, 0.8380)
DecisionTree	0.6059 ± 0.1392	(0.4331, 0.7788)
Randomforest	0.7086 ± 0.0769	(0.6132, 0.8041)
GradientBoosting	0.6146 ± 0.1603	(0.4157, 0.8136)

### 4.5 Model training

Model training is to build a classifier, that is, the process of model training and parameter tuning according to the previously selected data sets, features and algorithms, and its output is the built model. In this paper, a 5-fold cross-validation method is used for parameter tuning. Its basic idea is to group the training sets in the original data set. One part is used as the training set to train the model, and the other part is used as the test set to evaluate the model.

**Discriminant model**: as shown in Table 7, we selected totally 156 patients and normal people as the training set to build the discriminant model. For three different algorithms, the relevant parameters obtained are: KNN parameter n_neighbors = 6, SVM parameter c = 2.1, max_iter = 21, kernel = poly, Randomforest parameter n_ estimators = 9.

**Table 7:** Data of experiment 1.

Category	Number of subjects	Age	Height	Weight	Training set	Test set
Normal healthy people	187	47.4 ± 12.4	165.2 ± 7.4	69.7 ± 17.6	129	58
Stroke patients	39	63.4 ± 14.5	169.3 ± 7.1	77.5 ± 7.5	27	12

**Hierarchical model**: as shown in Table 8, we selected 32 patients with a total of 515 steps as our total sample, of which the training set is composed of 25 patients with a total of 457 steps to build the hierarchical model. For three different algorithms, the relevant parameters obtained are: Adaboost parameter n_estimators = 7, learning_ rate = 1.8, SVM parameter C = 0.1, max_iter = 7, kernel = poly, Randomforest parameter n_ estimators = 52.

**Table 8:** Data of experiment 2.

Category	Number of subjects	Number of steps	Training set (Steps / numbers of people)	Test set (Steps / numbers of people)
(0–1) very mild stroke	15	295	264/12	31/3
(2–5) mild stroke	17	220	193/13	27/4

## 5 Experimental results

Based on the trained discriminant model and hierarchical model for stroke gait analysis, 226 cases of data (including normal healthy people and stroke patients) were collected from Beijing Chao-Yang Hospital, Capital Medical University, and the models trained in this paper were tested to verify the effectiveness. The experiment is divided into two parts. The first part (hereinafter referred to as **Experiment 1**) is to verify the discriminant model, that is, to distinguish between normal healthy people and stroke patients, and the second part (hereinafter referred to as **Experiment 2**) is to verify the hierarchical model, that is, to judge the severity of stroke in stroke patients.

### 5.1 Experimental data

The details of the subjects participating in the experiment are shown in Table 7. This data is used as the data set of **Experiment 1**.

In Table 7, 32 of 39 stroke patients were scored and labeled by clinicians with NIHSS. The scores of the labeled patients are distributed between 1 and 5 points, that is, all patients are non-severe stroke patients, as shown in Table 9. During the test, the patients were required to walk on a flat ground along a straight line. According to the actual situation of the patients, the patients should walk as many times as possible, that is, different patients walked at different times. Each walk covered about 2–50 steps.

**Table 9:** NIHSS score of strokes.

NIHSS score / point	1	2	3	4	5	Total
Number / person	15	10	5	1	1	32

In this paper, we calculated the parameters of each step in each walk of each patient to form the data set of **Experiment 2**. The total number of steps of 32 patients was 515. In consideration of the symmetry of the sample, we classified the patients with a NIHSS score of 1 as very mild stroke, and the patients with a NIHSS score of 2–5 as mild stroke. The specific experimental data of **Experiment 2** are shown in Table 8.

### 5.2 Experimental results

The purpose of the experiment is to verify the performance of the discriminant model based on machine learning to distinguish between normal healthy people and stroke patients, and the performance of the hierarchical model to distinguish the severity of stroke in stroke patients.

(a) Experiment 1

Test results: as shown in Table 7, we selected 70 patients and normal people as test sets to verify the performance of the model. The experimental results are shown in Table 10. From the table:

**Table 10:** Experiment 1: test results by model comparing between different algorithms.

Algorithm	Average Accuracy	Stroke patients				Normal control			
		Total	Correct	Error	Accuracy	Total	Correct	Error	Accuracy
KNN	92.86%	12	8	4	66.67%	58	57	1	98.28%
SVM	92.86%	12	8	4	66.67%	58	57	1	98.28%
Random forest	90.00%	12	7	5	58.33%	58	56	2	96.55%

1. The model based on KNN, SVM and Randomforest algorithms has achieved 98.28, 98.28 and 96.55% detection accuracy for normal healthy people, respectively.
2. The detection accuracy of the model based on KNN, SVM and Randomforest algorithms for stroke patients was 66.67, 66.67 and 58.33%, respectively.
3. The overall detection accuracy of the model based on KNN, SVM and Randomforest algorithms is 92.86, 92.86 and 90.00%, respectively.

(b) Experiment 2

Test results: as shown in Table 8 we selected 32 patients with a total of 515 steps as our total sample. The test set consists of 7 patients with a total of 58 steps to verify the performance of the model. It should be noted that the judgment criterion of this experiment is: each prediction result is the prediction result of a certain step for a person. If the prediction result of each step is consistent with the labeled data, it is judged that the recognition is correct, otherwise it is a recognition error. The same judgment is made on all the steps of the person, and finally the principle of the minority obeying the majority is adopted. In other words, the total number of steps judged to be correct is more than the number of steps judged to be wrong, then the total judgment result is that the detection is correct; Otherwise, it is a detection error. The experimental results are shown in Table 11. Subject/tester 1–4 are mild stroke patients, and subject/tester 5–7 are very mild stroke patients. It can be seen from the table that the overall detection accuracy of the model based on Randomforest, SVM and AdaBoost algorithms reaches 71.43, 85.71 and 85.71%, respectively.

**Table 11:** Experiment 2: test results by model comparing between different algorithms.

Random forest		Number of steps	Correct steps detected	Error steps detected	Judged to be correct
Mild stroke	Tester 1	2	2	0	yes
	Tester 2	7	3	4	no
	Tester 3	11	0	11	no
	Tester 4	7	7	0	yes
Very mild stroke	Tester 5	8	7	1	yes
	Tester 6	7	6	1	yes
	Tester 7	16	16	0	yes
Total	7	58	41	17	5
Accuracy	71.43%				
SVM		Number of steps	Correct steps detected	Error steps detected	Judged to be correct
Mild stroke	Tester 1	2	2	0	yes
	Tester 2	7	4	3	yes
	Tester 3	11	2	9	no
	Tester 4	7	7	0	yes
Very mild stroke	Tester 5	8	6	2	yes
	Tester 6	7	4	3	yes
	Tester 7	16	16	0	yes
Total	7	58	41	17	6
Accuracy	85.71%				
Adaboost		Number of steps	Correct steps detected	Error steps detected	Judged to be correct
Mild stroke	Tester 1	2	2	0	yes
	Tester 2	7	4	3	yes
	Tester 3	11	3	8	no
	Tester 4	7	7	0	yes
Very mild stroke	Tester 5	8	7	0	yes
	Tester 6	7	5	2	yes
	Tester 7	16	11	5	yes
Total	7	58	39	18	6
Accuracy	85.71%				

## 6 Discussion

In this paper, a quantitative evaluation method of stroke association based on multi-dimensional gait parameters is proposed. This method is based on machine learning method, training a model, and then analyzing gait of stroke. That is, through all existing gait features (gait parameters), select appropriate gait features (gait parameters), label stroke patients with NHISS scale by clinicians, and finally train a discriminant model for stroke patients or not and a hierarchical model for stroke severity grading evaluation through machine learning algorithm. Based on the data of 226 cases collected in Beijing Chao-Yang Hospital, Capital Medical University, experiments were conducted on the two models. The purpose of **Experiment 1** was to use the discriminant model to distinguish between normal healthy people and stroke patients. The experimental results show that the three algorithms have achieved 96.55–98.28% detection accuracy for normal people, which shows that the classifier can better distinguish healthy people. The accuracy of the model based on KNN, SVM and Randomforest is 58.33–66.67% for stroke patients. Compared with healthy people, the accuracy of stroke patients’ detection is low. This is because the NIHSS scores of stroke patients in our test set are small, that is, the degree of stroke of these patients is very slight, so the classifier is difficult to distinguish under the current training of relatively small data sets. The overall detection accuracy of the models by the three algorithms has reached more than 90%, and the results of KNN and SVM detection are better, which fully shows that the model built in this paper can provide a certain degree of early warning for the onset or recurrence of stroke. The purpose of **Experiment 2** was to use the hierarchical model to distinguish the severity of stroke in stroke patients. It can be seen from the result that the overall detection accuracy of the models is up to 85.71% according to the number of steps, of which the results of AdaBoost and SVM are better, which fully shows that the model built in this paper can monitor the rehabilitation status of stroke patients after stroke. However, because the overall sample size of our experiment is not too large, and the selected patients have mild stroke, it has limitations. In the future, we need to expand the sample size for further in-depth research. In addition, previous studies have shown that the presence and progression of cerebral atrophy(subtype of ischemic stroke) is a potentially relevant (although still poorly characterized) manifestation of acute cerebral small vessel disease-related to gait disturbances (Grau-Olivares et al., 2010; Smith and Arboix, 2012). Because the pathophysiology, prognosis and clinical characteristics of acute small vessel ischemic stroke are different from other types of cerebral infarction, the two models in the proposed method for fine-grained domain of ischemic stroke is also one of the future research directions. For example, it can be used to study the relationship between lacunar and non-lacunar acute stroke (Rudilosso et al., 2022).

## 7 Conclusion

The two experimental results showed that the stroke association quantitative evaluation method by using machine learning based on multi-dimensional gait parameters proposed in this paper has the characteristics of high accuracy, objectivity, and quantification. It is expected to be used in clinical early warning, rehabilitation monitoring and evaluation of post-stroke recurrence to assist clinicians more efficiently.

## Data Availability

The raw data supporting the conclusions of this article will be made available by the authors, without undue reservation.
